# Immunohistochemical expression of insulin-like growth factor binding protein-3 in invasive breast cancers and ductal carcinoma *in situ*: implications for clinicopathology and patient outcome

**DOI:** 10.1186/bcr963

**Published:** 2004-11-23

**Authors:** Sarah B Vestey, Claire M Perks, Chandan Sen, Caroline J Calder, Jeff MP Holly, Zoe E Winters

**Affiliations:** 1 University of Bristol, Department of Clinical Sciences at South Bristol – Surgery, Bristol Royal Infirmary, Bristol, UK; 2 Department of Histopathology, United Bristol Healthcare NHS Trust, Bristol Royal Infirmary, Bristol, UK

**Keywords:** clinicopathology, ductal carcinoma *in situ*, IGFBP-3, immunohistochemistry, prognosis

## Abstract

**Introduction:**

Insulin-like growth factor binding protein-3 (IGFBP-3) differentially modulates breast epithelial cell growth through insulin-like growth factor (IGF)-dependent and IGF-independent pathways and is a direct (IGF-independent) growth inhibitor as well as a mitogen that potentiates EGF (epidermal growth factor) and interacts with HER-2. Previously, high IGFBP-3 levels in breast cancers have been determined by enzyme-linked immunosorbent assay and immunoradiometric assay methods. *In vitro*, IGFBP-3's mechanisms of action may involve cell membrane binding and nuclear translocation. To evaluate tumour-specific IGFBP-3 expression and its subcellular localisation, this study examined immunohistochemical IGFBP-3 expression in a series of invasive ductal breast cancers (IDCs) with synchronous ductal carcinomas *in situ *(DCIS) in relation to clinicopathological variables and patient outcome.

**Methods:**

Immunohistochemical expression of IGFBP-3 was evaluated with the sheep polyclonal antiserum (developed in house) with staining performed as described previously.

**Results:**

IGFBP-3 was evaluable in 101 patients with a variable pattern of cytoplasmic expression (positivity of 1+/2+ score) in 85% of invasive and 90% of DCIS components. Strong (2+) IGFBP-3 expression was evident in 32 IDCs and 40 cases of DCIS. A minority of invasive tumours (15%) and DCIS (10%) lacked IGFBP-3 expression. Nuclear IGFBP-3 expression was not detectable in either invasive cancers or DCIS, with a consistent similarity in IGFBP-3 immunoreactivity in IDCs and DCIS. Positive IGFBP-3 expression showed a possible trend in association with increased proliferation (*P *= 0.096), oestrogen receptor (ER) negativity (*P *= 0.06) and HER-2 overexpression (*P *= 0.065) in invasive tumours and a strong association with ER negativity (*P *= 0.037) in DCIS. Although IGFBP-3 expression was not an independent prognosticator, IGFBP-3-positive breast cancers may have shorter disease-free and overall survivals, although these did not reach statistical significance.

**Conclusions:**

Increased breast epithelial IGFBP-3 expression is a feature of tumorigenesis with cytoplasmic immunoreactivity in the absence of significant nuclear localisation in IDCs and DCIS. There are trends between high levels of IGFBP-3 and poor prognostic features, suggesting that IGFBP-3 is a potential mitogen. IGFBP-3 is not an independent prognosticator for overall survival or disease-free survival, to reflect its dual effects on breast cancer growth regulated by complex pathways *in vivo *that may relate to its interactions with other growth factors.

## Introduction

Insulin-like growth factors (IGF-I and IGF-II) regulate the cellular growth of normal and malignant breast epithelial cells with a role in malignant transformation [1-3]. IGFs are potent mitogens and are synergistic with oestrogen and epidermal growth factor (EGF) to stimulate cellular proliferation[B4]. The IGFs are modulated by a family of six high-affinity IGF binding proteins, of which IGFBP-3 predominates in serum and is upregulated in breast cancer cell lines, including breast epithelium [1,5,6]. Both IGFs (IGF-I and IGF-II) have a preferential stromal expression and together with epithelial IGFBP-3 have a significant paracrine influence on breast epithelial growth [1,7]. IGFBPs have multiple and complex functions that can be either IGF dependent or IGF independent. With respect to IGF-dependent function, IGFBP-3 preferentially binds IGFs to either inhibit or activate IGF mitogenic effects *in vitro*, through blocking the IGF-receptor interaction, in contrast to a prolongation of IGFs' half-life and their protection from degradation [1-3]. IGFBP-3 is pro-apoptotic in an IGF-dependent manner, as well as an IGF-independent manner, and enhances the p53 DNA damage response *in vitro *[8,9]. The particular significance of IGFBP-3 in regulating epithelial cell growth has been highlighted because the actions of many growth inhibitors, apoptotic agents and anti-cancer treatments (transforming growth factor-β, retinoids, p53 and anti-oestrogens) are, at least in part, mediated by their ability to stimulate local IGFBP-3 production [1-3].

Through complex and as yet poorly understood mechanisms, IGFBP-3 is a direct (IGF-independent) growth inhibitor as well as a mitogen on breast epithelial cells[B10,^11^]. Further, IGFBP-3 inhibits oestradiol-stimulated cell proliferation in breast cancer cell lines, with the potential to accentuate ceramide and paclitaxel-induced apoptosis directly [12-14]. By contrast, accumulating evidence *in vitro *suggests the potential mitogenicity of IGFBP-3 through its interactions with EGF receptor (EGFR) and Ras-p44/42 mitogen-activated protein kinase (MAPK) signalling in breast epithelial cells [15-18]. Postulated mechanisms for IGFBP-3's direct intrinsic actions on cell growth and apoptosis have not as yet characterised a definitive cell membrane receptor or the necessity for IGFBP-3 cell surface interaction or nuclear translocation[B19]. Intracellular trafficking of IGFBP-3 with nuclear localisation in T47D breast cancer cells is explicable through a carboxy-terminal nuclear localisation signal and importin-β-mediated nuclear transport[B15,^20^,^21^]. At present, the functional implications of nuclear IGFBP-3 are unknown[B22].

Complex IGFBP-3 modulation of breast cancer growth has prompted several studies to examine levels of IGFBP-3 in breast cancer tissues in relation to clinicopathological characteristics and patient outcome [23-26]. Circulating IGFBP-3 may avert breast cancer development, with the clinical paradox that increasing IGFBP-3 levels in breast tumours may indicate adverse prognostic cancers [2,23-26]. This reflects the complexity of IGFBP-3 effects on cell proliferation and its potential role as a mitogen as well as a growth inhibitor. Poor prognostic tumours with increasing IGFBP-3 expression may relate to recent evidence *in vitro *in which the pro-apoptotic action of IGFBP-3 is reversed by the extracellular matrix protein fibronectin[B27,^28^]. In keeping with these findings, high levels of fibronectin expression are associated with poor prognostic breast cancers [6,29]. IGFBP-3 interacts with integrin-receptor signalling with modulation by fibronectin to increase cell attachment and possible resistance to apoptosis[B27].

The aim of this first immunohistochemical study was to evaluate breast epithelial IGFBP-3 expression in relation to clinicopathological parameters and prognosis in breast cancer. IGFBPs are upregulated in malignant breast epithelial cells with evidence *in vivo *that IGFBP-5 is overexpressed in the cytoplasm of breast cancers and their lymph node metastases on tissue microassay immunohistochemistry (IHC)[B6]. Accumulating evidence *in vitro *supports the dual effects of IGFBP-3 on the cellular growth of breast epithelial cells to emphasise the importance of selectively analysing breast epithelial IGFBP-3 expression in comparison with the stroma. By contrast, previous studies have collectively assessed breast epithelial and stromal IGFBP-3 levels by using immunoassay and immunoblot or ligand blot methods [23-26]. Moreover, both the histological location of IGFBP-3 and variations in its immunoreactivity have been compared in this series of invasive ductal breast cancers with concomitant evaluation of IGFBP-3 expression in synchronous ductal carcinoma *in situ *(DCIS) within the same tumour specimens. Increasing IGFBP-3 levels in breast tumours may indicate adverse prognostic cancers, with contradictory implications on patient outcomes[B24,^25^] to reflect the complexity of IGFBP-3 effects on cell proliferation[B27,^28^,^30^]. We have shown a varying pattern of epithelial IGFBP-3 cytoplasmic expression (1+/2+ score) in 85% of invasive ductal cancers (IDCs) and 90% of DCIS components, without detectable nuclear immunoreactivity. Increasing levels of IGFBP-3 expression showed a trend with increased proliferation, oestrogen receptor (ER) negativity and HER-2 overexpression to suggest its association with poor prognostic tumours.

## Methods

### Patients

The study included 103 patients aged from 26 to 88 years (median 59 years) with IDC of the breast, in association with concomitant DCIS diagnosed between 1996 and 2000 at the Bristol Royal Infirmary, Bristol, UK (Table 1). Patient numbers in clinicopathological subgroups reflect those in whom IGFBP-3 was evaluable. Regional Ethics Committee approval was granted before the start of the study. Axillary lymphadenopathy was evaluable in 88 patients; 37 (42%) were lymph-node-negative and 51 (58%) were lymph-node-positive (N1, mobile ipsilateral, or N2, fixed ipsilateral) patients. No axillary surgery was undertaken in the remaining 13 patients because of age-related co-morbidity. Clinicopathological subgroups were analysed in accordance with the Nottingham Prognostic Index (NPI) and divided into good (GPG), moderate (MPG) and poor (PPG) prognostic groups as described, with a modification that included no assessment of the internal mammary lymph nodes[B31]. Evaluation of the NPI was precluded in 15 patients because of non-evaluable regional lymphadenopathy and tumour size. Similarly, subgroups of DCIS were analysed in accordance with the Van Nuys Pathologic Classification (VNPC) (Table 1), which was not assessable in five patients [32]. The design of the study to include tumour representative samples of synchronous IDC and DCIS precluded the analysis of the Van Nuys Prognostic Index[B32]. Adjuvant treatment groups comprised the following: tamoxifen in 60 patients (27 GPG, 16 MPG, 4 PPG and 13 no NPI), and CMF-containing and anthracycline-containing regimes in 17 and 21 patients, respectively (4 GPG, 19 MPG, 13 PPG and 2 no NPI). Five patients received no adjuvant treatment. The median follow-up duration was 51 months (range 4–120 months). All were primary tumours with the exception of six local tumour recurrences, which were excluded from the analysis of patient outcome (see Table 3 and Fig. 2).

Tumour samples were collected and freshly fixed in buffered formalin in accordance with a standardised protocol at a single institution. Tumours were classified in accordance with NHSBSP guidelines [33]. Invasive ductal carcinomas were graded by the modified Bloom's grading system described by Elston and Ellis[B34]. ER immunostaining was performed with a standard three-layered streptavidin-avidin-biotin horseradish peroxidase method with a mouse anti-human ER primary antibody (M0747, 1:100 dilution; DAKO, Ely, Cambridgeshire, UK) and a biotinylated rabbit anti-mouse secondary antibody (E354, 1:350 dilution; DAKO). Expression of ER was assessed with the quick-score (0–8) and classified as positive (4–8; >3) or negative (0–3; ≤ 3) in five high-power fields (HPFs) [35]. Tumour proliferation was assessed with nuclear Ki67 immunostaining (polyclonal rabbit anti-human Ki67 antigen; A0047, 1:100 dilution; DAKO). A goat anti-rabbit biotin-labelled polypeptide (E432, 1:400 dilution; DAKO, Glostrup, Denmark) was used as a secondary antibody.

Tonsillar tissue was used as a positive control and primary antibody was replaced with Tris-buffered saline (TBS) as a negative control. Ki67 staining was evaluated as percentage of positive tumour cells, with low proliferation indicative of <10% of positive-staining cells, compared with high proliferation with ≥ 10% positivity [36]. HER-2 immunostaining was performed with the mouse monoclonal anti-HER-2 antibody (RTU-CB11; Novocastra/Vector, Newcastle upon Tyne, UK), and the Envision Plus HRP system (K4006; DAKO). HER-2 expression was scored according to the degree and proportion of membrane staining, with a score of 0 or 1+ defined as negative, and 2+ or 3+ as HER-2 positive[B37]. Lymphovascular invasion was assessed as present or not, and together with ER, HER-2 and Ki67 was analysed in the Department of Pathology (by CS and CC).

### Immunohistochemistry

IGFBP-3 immunoreactivity was evaluated with the in-house sheep polyclonal antiserum (Professor JMP Holly, IGF Research Group, University of Bristol, Bristol, UK) at 1:800 dilution[B9]. IGFBP-3 immunostaining of IDC and DCIS was compared with formalin-fixed normal liver tissue as a positive control for IGFBP-3, and a replacement of the primary antibody with TBS as a negative control. Validation and specificity of the sheep polyclonal in-house antiserum has previously been demonstrated with an IGFBP-3 peptide on immunocytochemistry of Hs578T breast cancer cells[B38].

Formalin-fixed paraffin sections of breast cancer tissue and normal liver tissue were mounted on glass slides coated with 3-aminopropyl-triethoxysilane (APES; Sigma, Poole, Dorset, UK) and were baked for 30 min at 56–60°C, before being dewaxed in Clearene (Surgipath Europe, Peterborough, UK). The tissue was rehydrated by sequential immersion in 100% and 50% ethanol to distilled water. Tissue sections were subjected to heat antigen retrieval for 3 min in citrate buffer (pH 6) in a pressure cooker, and after cooling were incubated for 5 min in 0.3% (v/v) hydrogen peroxide. Subsequently, sections were washed in tap water and TBS (pH 7.45). Before incubation with IGFBP-3 primary antibody, sections were exposed to avidin and biotin blocking solutions (Vector Laboratories, Burlingame, CA, USA) for 15 min, respectively. Further blocking was achieved through exposure to normal rabbit serum (diluted with TBS) for 30 min at room temperature (20–22°C). Primary antibody was applied and incubated overnight at 4°C (18 hours). After washing with TBS, biotinylated rabbit anti-goat secondary antibody, together with the Strept-AB Complex/HRP (0377, DAKO, Glostrup, Denmark) was applied for 30 min at room temperature. Staining was revealed by development in the chromogen 3,3-diaminobenzidine tetrahydrochloride (DAB) for 5–10 min before counterstaining with haematoxylin in preparation for mounting.

Immunostaining was assessed with a Zeiss Axioskop microscope with a 40× Achrostigmat lens (× 400 overall magnification) and a field diameter of 0.46 mm. In the neoplastic cell population for IDC and DCIS, the degree of staining intensity and the proportion of cells with IGFBP-3 immunoreactivity in the nucleus and cytoplasm were graded semi-quantitatively to produce an intensity distribution score for each localisation, with invasive and pre-invasive components given separate scores. Initial scoring was of 10 HPFs; however, in view of the homogeneous staining, this was reduced to 5 HPFs. Sections were scored independently by two observers and were scored as follows: negative (0), weak/moderately positive (1+) or strongly positive (2+), with DCIS and invasive components scored independently. Scores were assessed as a continuum for the purposes of statistical correlation, unless otherwise stated.

### Statistical analysis

Data were analysed with the SPSS 10.0 for Windows statistics software and summarised with descriptive statistics. The associations between IGFBP-3 and patient characteristics were assessed with the Spearman non-parametric test for continuous variables and the χ^2 ^test for categorical factors. Analyses of survival data were performed with the log-rank test and the Cox regression model, and survival curves were computed with the Kaplan-Meier method. For IGFBP-3, univariate and multivariate analyses were performed, the latter adjusting for NPI score and treatment received (tamoxifen/chemotherapy/none). Because the NPI is based on nodal involvement, on tumour size and on grade, patients (*n *= 11) with non-evaluable lymphadenopathy and tumour size were excluded from the multivariate regression analyses (see Table 3).

## Results

### IGFBP-3 expression in IDCs and DCIS and their relationships to clinicopathological factors

**Figure 1:**
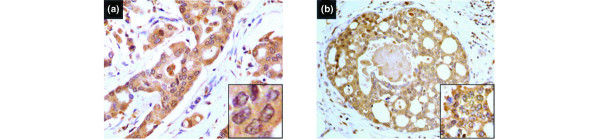
Examples of IGFBP-3 immunoreactivity in infiltrating ductal carcinoma of the breast with concomitant DCIS. Immunostaining was performed as described in the Methods section, and nuclei were counterstained with haematoxylin. **(a) **A tumour showing cytoplasmic IGFBP-3 expression in invasive ductal cancer. **(b) **A similar cytoplasmic IGFBP-3 positivity in concomitant DCIS. High-power magnification (original magnification × 400).

IGFBP-3 immunoreactivity was evaluable in 101 (98%) cases of IDCs and in 102 cases of DCIS, and scored positively (1+/2+) as a homogeneous cytoplasmic expression in 86 (85%) of invasive and 92 (90%) of DCIS components (Fig. 1; Tables 1 and 2). Strong (2+) IGFBP-3 expression was seen in 32 invasive breast cancers and 40 cases of DCIS. A weak/moderate (1+) expression of IGFBP-3 was evident in the majority of invasive (*n *= 54) and DCIS (*n *= 52) components. There was no clear evidence of nuclear IGFBP-3 expression on IHC in IDC or DCIS. Consistently low levels of IGFBP-3 were evident throughout the stroma, without strong expression except in vascular endothelial cells. A comparison showed that the levels of IGFBP-3 expression in DCIS were similar to those in invasive disease (Table 2).

We investigated the relationships between the levels of IGFBP-3 expression and clinicopathological parameters in IDCs and DCIS. IGFBP-3 scores were analysed as a continuum for the purposes of statistical analysis. There were no significant associations (where data was assessed as a continuum) between IGFBP-3 and established prognostic indicators in invasive disease (lymph node involvement, increasing tumour size, increasing tumour histological grade, ER negativity [quick-score 0–8], lymphovascular invasion and NPI). There was a possible trend between increasing IGFBP-3 levels and increasing cellular proliferation (Ki67) in invasive disease (Spearman correlation coefficient [cc] 0.166, *P *= 0.096) (where assessed as a continuum) that was not observed in DCIS (*P *= 0.8) (data not shown), or where Ki67 was categorised as <10% versus ≥ 10% (Table 1). A comparison of categorical IGFBP-3 expression with ER-positive (quick-score 4–8) and ER-negative (quick-score 0–3) IDCs showed a trend (*P *= 0.06) with ER-negative tumours and HER-2-positive invasive tumours (*P *= 0.065) (Table 1). In Table 1, 94% (33 of 35) of ER-negative cancers expressed either 1+ or 2+ IGFBP-3 versus 80% of ER-positive tumours (χ^2 ^test, *P *= 0.06). Similarly, 94% (15 of 16) of HER-2-positive tumours expressed IGFBP-3 (1+/2+) versus 84% of HER-2-negative cancers (*P *= 0.065).

There were no associations between IGFBP-3 expression and pathological variables on logistic regression in DCIS (VNPC, HER-2 expression and increased proliferation/Ki67). A categorical analysis of individual IGFBP-3 scores (negative versus 1+ versus 2+) in DCIS showed that 97% (32 of 33) of IGFBP-3-positive DCIS were ER-negative versus 80% of ER-positive DCIS (*P *= 0.037) (Table 1). We demonstrated an inverse correlation between local IGFBP-3 expression and patient age, in which IGFBP-3 immunoreactivity analysed on a continuum decreased with age (cc -0.214, *P *= 0.03) (data not shown); Table 1 shows a significant association with age (*P *= 0.04) when IGFBP-3 and age are analysed categorically in the invasive components, with a possible trend in DCIS (cc -0.174, *P *= 0.08) (data not shown).

### Relationships of clinicopathological factors to prognosis and the predictive potential of IGFBP-3 expression

Overall survival (OS) and disease-free survival (DFS) were determined in 87 and 95 patients, respectively, with a median follow-up of 51 months (range 4–120 months). Disease relapses (local or distant recurrences) occurred in 30 women; of these, deaths were confirmed in 23 patients, with 8 suspected deaths in the absence of a recorded mortality date. Locoregional recurrence occurred at a median duration of 28.5 months (range 3–156 months) from diagnosis. Breast cancer-related mortality occurred at a median of 26 months (range 8–98 months) from presentation. The mean durations of OS and DFS were 93 months and 87 months, respectively. Four-year DFS and OS were 70% and 77%, respectively. The relationship of established clinicopathological features with OS and DFS were analysed with Cox's regression analysis (Table 3). Generally poor prognostic factors such as large tumour size, high tumour grade, lymphovascular invasion, lymph node metastases, ER negativity, HER-2 overexpression and NPI were significantly associated with decreased OS and DFS. High tumour proliferation (Ki67 on IHC), although associated with a lower percentage of patients remaining disease-free (DFS) and alive (OS) at 4 years, did not reach statistical significance.

**Figure 2:**
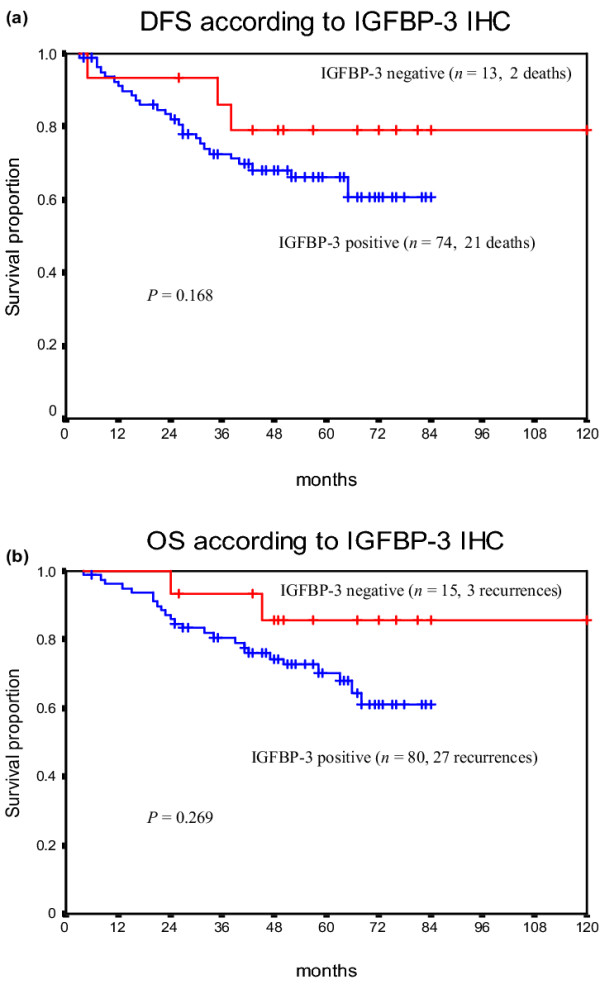
The relationship of IGFBP-3 to disease-free survival (DFS) and overall survival (OS). DFS **(a) **and OS **(b) **curves according to IGFBP-3-positive (1+/2+) and IGFBP-3-negative (0) breast cancers. The patient numbers reflect the exclusion of six local tumour recurrences as described in the Methods section, and include only recorded deaths in 23 patients. The *P *values are given for the log ranks.

Univariate and multivariate analysis with the continuous score variables were used to investigate possible relationships between patient outcome data and levels of expression for IGFBP-3. IGFBP-3 scores (assessed as a continuum) were not predictive for OS or DFS after univariate or multivariate analysis (Table 3), adjusted for NPI and adjuvant treatment (tamoxifen/chemotherapy/none) with respect to the multivariate analysis. Where IGFBP-3 was categorised as positive (1+/2+) or negative (0), the Kaplan-Meier survival curves (Fig. 2) demonstrate a possible trend towards a more favourable outcome for IGFBP-3-negative tumours, although this failed to reach statistical significance for OS (*P *= 0.168) or DFS (*P *= 0.269), perhaps related in part to the small numbers of IGFBP-3-negative tumours observed in this study.

## Discussion

IGFBP-3 has direct intrinsic actions, as well as regulating IGFs to influence cellular growth, survival and apoptosis of breast epithelial cells. Breast cancer cells typically express several IGFBPs, with IGFBP-2, IGFBP-3, IGFBP-4 and IGFBP-5 being observed most often [5,6,22]. Although previous studies *in vivo *have suggested a tumour-related upregulation of IGFBP-3 levels, clinical studies have yet to determine IGFBP-3 epithelial protein expression in breast cancers; this is the first IHC study. IHC of tissue microassays confirms a tumour-specific upregulation of IGFBP-5 and IGFBP-2 in primary breast cancers and their lymph node metastases [6]. The significance of these findings in the context of a decreased mRNA expression for IGFBP-5 and IGFBP-2, respectively, highlights the value and clinical contribution of an immunohistochemical evaluation[B6]. Furthermore, a tumour-specific pathway is implicated, with negligible levels of IGFBP-5 and IGFBP-2 in normal breast epithelial cells[B6,^39^,^40^]. Increasing levels of IGFBP-3 in breast cancer tissues correlate with poor prognostic features, as demonstrated in four studies *in vivo *through enzyme-linked immunosorbent assay and immunoradiometric assay [23-26]. The admixture of tumour with normal and stromal breast tissue is a feature of both quantitative methods with possible limitations regarding the evaluation of a predominant tumour-specific epithelial protein.

No studies have yet clarified IGFBP-3 expression on IHC in invasive breast cancers in comparison with DCIS, although there has been a limited review of colorectal carcinomas and normal colonic mucosa[B9]. This study suggests that most breast cancers express epithelial IGFBP-3 (1+/2+), mostly with a weak/moderate immunoreactivity (1+). Fewer tumours expressed IGFBP-3 strongly (2+) and it was evident more frequently in DCIS. Similar genomic aberrations may occur in DCIS and IDC to highlight possible similarities in protein expression [41,42]. This study, in large part, shows a consistent similarity in the levels of IGFBP-3 expression in DCIS and IDC (cc 0.789; *P *< 0.001) (Table 2).

Nuclear localisation of IGFBP-3 has been described in several cell lines *in vitro*, including breast cancer cells[20,21]. This observation is supported by the direct interaction of IGFBP-3 with the nuclear receptor retinoid X receptor and the ability of IGFBP-3 to act as a nuclear-import carrier for IGF-I[B20,^22^,^43^]. This has raised considerable interest in the potential nuclear actions of IGFBP-3 and its functional implications. There have been very few observations of nuclear localisation of IGFBPs *in vivo*, with cytoplasmic IGFBP-5 expression in breast tumours and, similarly, cytoplasmic IGFBP-3 in normal colonic crypts[B6,^9^]. Moreover, there is further evidence *in vitro *suggesting that IGFBP-3 growth modulation might be independent of nuclear translocation[B19]. In the 101 samples of IDC and DCIS analysed in the study, we observed no evidence of nuclear staining for IGFBP-3.

IGFBP-3 modulates cellular proliferation with dual actions that either enhance IGFs or inhibit their actions. By contrast, in IGF-unresponsive Hs578T breast cancer cells, IGFBP-3 is predominantly growth-inhibitory and pro-apoptotic[B11,^27^]. Similarly, there is a potential for IGFBP-3 to switch its action on cell survival in Hs578T cells through changes in the extracellular matrix, with a clear reversal of IGFBP-3 accentuation of apoptosis when cells are changed from being grown on either plastic, collagen or laminin to fibronectin[B27]. This suggests that IGFBP-3 might be preferentially activating integrin receptors that bind fibronectin in a pro-survival growth stimulatory pathway[B44,^45^]. Upregulation of fibronectin expression is a feature of breast cancer metastases and poor prognostic tumours, with evidence of IGFBP-3 binding to this mesenchymal extracellular matrix glycoprotein[B29,^46^,^47^]. Tumour-related upregulation of IGFBP-3 levels in aggressive breast cancers is only partly explicable by the described findings *in vitro*.

Evidence is accumulating that IGFBP-3 is a potential mitogen that interacts with EGFR and HER-2 signalling pathways [16-18,28]. The potential to switch the IGFBP-3 action on cell growth suggests that IGFBP-3 has a role in malignant progression of breast cancer cells with insensitivity to IGFBP-3 growth inhibition through the expression of oncogenic Ras [17]. This is further supported by four clinical studies of breast tumours, including the present study demonstrating a spectrum of increased IGFBP-3 levels, with the highest expression indicative of a more malignant phenotype [23-26]. Increasing passages of T47D cells switch their response to IGFBP-3 with increasing tumorigenicity, although initially growth-inhibited by this binding protein [18]. Dual growth modulation by IGFBP-3 is demonstrated in MCF10A cells, in which IGFBP-3 changes from a growth inhibitor to a mitogen through Ras-induced malignant transformation and activation of Ras-p44/42 MAPK [16,17]. Normal breast epithelial MCF10A cells exposed to increasing doses of IGFBP-3 show a similar biphasic response, with preliminary growth inhibition followed by IGFBP-3 mitogenicity in the context of an IGF-I receptor antagonist, or a serine phosphorylation domain peptide (SPD, a non-IGF binding peptide), to verify these IGF-independent effects[B11,^48^]. LNCaP prostate cancer cells are similarly growth-stimulated by IGFBP-3 independently of IGFs [49].

In this study we found a possible association with increased proliferation, together with other adverse prognostic features such as ER negativity and HER-2 overexpression. Elevated IGFBP-3 expression might therefore suggest an abundance of IGFs sequestered by the binding protein, with further implications of an IGF-independent mitogenic role. IGFBP-3 mitogenicity may also relate to candidate proteases, such as cathepsin D, prostate-specific antigen and matrix metalloproteinases, with IGFBP-3 proteolysis potentially releasing IGFs to enhance their mitogenicity. The precise mechanism for this regulation remains unknown because studies so far have shown no clear correlations between IGFBP-3 and these proteins in tissue extracts.

This study suggests an association between IGFBP-3 expression and ER negativity and confirms previous findings *in vivo *[24,25]. Clearly, there is a significant synergism between the ER and the IGFs in breast cancer cells [1-3]. Many members of the IGF system are under transcriptional control of the ER, with IGF-I similarly enhancing the transcriptional activity of ER[B1,^2^]. Oestrogens transcriptionally downregulate IGFBPs in breast tissue and increase IGFBP-3 proteases, which may in part explain the inverse association between IGFBP-3 and ER expression [24,25], as well as perhaps reflecting the disruption of common pathways characteristic of poor prognostic tumours [2,50,51]. HER-2 overexpression predicts aggressive and poor prognostic breast tumours that are likely to be ER negative and tamoxifen resistant[B52]. Recent evidence *in vitro *suggests a functional interaction between the IGF-I receptor and HER-2, with the potential for IGFBP-3 to modulate this response[B53,^54^]. Our finding of an association between IGFBP-3 and HER-2 expression is preliminary and, although based on a limited number of HER-2-positive tumours, may suggest a role for HER-2 in IGFBP-3 growth modulation. *In vitro*, IGFBP-3 promotes EGF in HER-2-overexpressing T47D and Hs578T breast cancer cells [18,28,30]. Ageing is associated with profound changes in the growth hormone/IGF regulatory pathways, with diminished circulating IGFBP-3, as well as decreased tissue levels previously observed *in vivo *and confirmed in this study [2,25].

Although high levels of IGFBP-3 in breast cancers are associated with poor prognostic features of the tumour, few studies have substantiated any significant implications on patient outcome[B24,^25^]. Tissue IGFBP-3 concentrations have been reported to predict a reduced OS, but this was not associated with breast cancer recurrence [25]. This study suggests that IGFBP-3 expression in breast cancers might be associated with a shorter OS and DFS, although few patients were negative for IGFBP-3 expression. Other studies, including the present one, show a consistent negative association between IGFBP-3 expression and favourable prognostic markers underlining the potential importance of this pathway in tumorigenesis [23-26]. IGFBP-3 regulates breast cancer epithelial growth through IGF-dependent and IGF-independent pathways that involve both growth inhibition and enhanced apoptosis with the potential to switch to growth stimulatory pathways interacting with EGFR, HER-2 and fibronectin. Defining these mechanisms merits further studies *in vitro *and *in vivo*.

## Conclusion

IGFBP-3 is important in tumorigenesis because of its effects on cellular proliferation, survival and apoptosis. We have demonstrated a tumour-associated upregulation of cytoplasmic IGFBP-3 epithelial expression in invasive and non-invasive breast cancers, with similar patterns of immunoreactivity. Despite evidence *in vitro *and functional implications, no nuclear IGFBP-3 expression was detectable on IHC. Invasive breast cancers expressing IGFBP-3 showed an association with poor prognostic features including increased proliferation, ER negativity and HER-2 overexpression, with possible implications for patient outcome. IGFBP-3 is a growth modulator with the potential to switch from a growth inhibitor to a mitogen that interacts with the EGFR family.

## Abbreviations

cc = Spearman correlation coefficient; DCIS = ductal carcinoma *in situ*; DFS = disease-free survival; ER = oestrogen receptor; GPG = good prognostic group; HPF = high-power field; IDC = invasive ductal cancer; IGF = insulin-like growth factor; IGFBP-3 = insulin-like growth factor binding protein-3; IHC = immunohistochemistry; MAPK = mitogen-activated protein kinase; MPG = moderate prognostic group; NPI = Nottingham Prognostic Index; OS = overall survival; PPG = poor prognostic group; TBS = Tris-buffered saline; VNPC = Van Nuys Pathologic Classification.

## Competing interests

The author(s) declare that they have no competing interests.

## Authors' contributions

SBV carried out the immunohistochemistry for IGFBP-3 as well as the clinical and statistical analyses. CMP established conditions for testing the IGFBP-3 antibody and together with JMPH has contributed to the development of the in-house antibody. CS evaluated all scoring of IGFBP-3 expression and performed all histopathology review. CJC evaluated the IGFBP-3 scoring and re-reviewed all histopathology. JMPH developed the IGFBP-3 antibody and conditions for its application, as well as conceiving the study. ZEW conceived the study, and participated in the study design, statistical analyses and wrote the manuscript. All authors read and approved the final manuscript.

## Figures and Tables

**Table 1:** Relationship between IGFBP-3 expression and clinicopathology

Characteristic	IGFBP-3 score			*P*	No. of patients
			
	Negative	Weak (1+)	Strong (2+)		
Invasive tumours	15	54	32		101*
Age (years)					
≤ 50	1	13	13	**0.04**	27
> 50	14	41	19		74
Lymph node status					
Negative	7	17	13	0.216	37
Positive	4	31	16		51
Not assessed					13
IDC grade					
I	2	9	5	0.836	16
II	9	24	17		50
III	4	21	10		35
IDC size					
≤ 2 cm	9	26	17	0.720	52
>2 cm	6	27	14		47
Multifocal					2
LV invasion					
Absent	9	27	12	0.411	48
Present	6	26	18		50
Not assessed					3
NPI					
GPG (< 3.4)	6	17	11	0.662	34
MPG (3.4–5.4)	4	19	13		36
PPG (> 5.4)	1	11	4		16
Not calculable					15
ER (quick-score)					
Positive (4–8)	12	28	21	0.06	61
Negative (0–3)	2	24	9		35
Not assessed					7
HER-2 IHC					
Negative (0/1+)	14	48	23	0.065	85
Positive (2+/3+)	1	6	9		16
Ki67 IHC					
Low proliferation < 10%	10	26	13	0.249	49
High proliferation ≥ 10%	5	28	19		52
DCIS					102*
VNPC					
Grade I	1	14	8	0.586	23
Grade II	5	17	14		36
Grade III	2	21	15		38
Not assessed					5
ER (quick-score)					
Positive (4–8)	11	26	19	**0.037**	56
Negative (0–3)	1	23	9		33
Not assessed					13
HER-2 IHC					
Negative (0/1+)	7	41	28	0.876	76
Positive (2+/3+)	2	9	8		19
Not assessed					7
Ki67 IHC					
Low proliferation < 10%	6	30	24	0.972	60
High proliferation ≥ 10%	4	22	16		42

DCIS, ductal carcinoma *in situ*; ER, oestrogen receptor; GPG, good prognostic group; IHC, immunohistochemistry; MPG, moderate prognostic group; NPI, Nottingham Prognostic Index; PPG, poor prognostic group; VNPC, Van Nuys Pathologic classification. Significant *P *values (*P *< 0.05) are indicated in bold.

*Patient numbers reflect those in whom IGFBP-3 was evaluable.

**Table 2:** Immunohistochemical (IHC) scores (0–2) for IGFBP-3 expression in the cytoplasm of invasive ductal cancers and ductal carcinoma *in situ*

Cancer type	Total no. of tumours	IGFBP-3 IHC score		
		
		Negative (0)	Weak/moderate (1+)	Strong (2+)
Invasive	101	15	54	32
DCIS	102	10	52	40

Tumour numbers scored (0–2) on immunohistochemistry (IHC) for IGFBP-3 expression in the cytoplasm of invasive cancers and ductal carcinoma *in situ *(DCIS). IHC expression was individually assessed in the nucleus and cytoplasm, and defined as negative (0), weak/moderate (1+) or strong (2+). Positivity for IGFBP-3 expression was defined as an IHC score of (1+/2+).

**Table 3:** Relationships between clinicopathological criteria, cytoplasmic IGFBP-3 and patient outcome

Variable	Univariate analysis		Multivariate analysis	
	95% CI	*P*	95% CI	*P*
Overall survival				
Age	0.99–1.04	0.4	0.98–2.90	**0.009**
Lymph node status (+/-)	2.79–153.9	**0.004**	2.65–161	**0.003**
Invasive tumour grade (I, II, III)	1.27–4.93	**0.002**	1.14–4.62	**0.002**
Tumour size	1.02–1.05	**<0.001**	1.02–1.05	**<0.001**
NPI	1.48–3.36	**<0.001**	1.39–3.32	**<0.001**
ER (quick-score)	0.66–0.92	**0.002**	0.68–0.98	**0.03**
HER-2 IHC score	0.94–2.02	**0.04**	0.76–1.95	0.56
Ki67 proliferative index	0.99–1.00	0.4	0.99–1.00	0.65
Lymphovascular invasion (+/-)	1.46–9.38	**0.006**	0.89–6.69	0.08
IGFBP-3 IHC score	0.62–2.01	0.719	0.59–2.50	0.592

Disease-free survival				
Age	0.97–1.02	0.9	0.99–1.06	0.06
Lymph node status (+/-)	2.29–25.3	**<0.001**	2.06–25.6	**0.002**
Invasive tumour grade (I, II, III)	1.48–5.38	**0.005**	1.36–5.11	**0.006**
Tumour size	1.02–1.04	**<0.001**	1.02–1.04	**<0.001**
NPI	1.46–3.17	**<0.001**	1.38–3.15	**<0.001**
ER (quick-score)	0.66–0.89	**0.001**	0.68–0.95	**0.01**
HER-2 IHC score	1.04–1.92	**0.02**	0.95–1.87	0.2
Ki67 proliferative index	0.99–1.00	0.2	0.99–1.00	0.3
Lymphovascular invasion (+/-)	1.63–6.95	**0.002**	1.09–6.65	**0.03**
IGFBP-3 IHC score	0.61–1.71	0.922	0.57–1.90	0.896

Confidence intervals (CI) and *P *values are given for the results of both the univariate and multivariate analyses. The multivariate analysis is adjusted for Nottingham Prognostic Index (NPI) (nodes, grade and size) and treatment (tamoxifen/chemotherapy/none). Data for univariate analysis were evaluable in 95 patients (reflecting the exclusion of six local tumour recurrences as described in the Methods section) and included a multivariate analysis on 84 cases that excluded non-evaluable NPI in 11 patients. All clinicopathological variables and cytoplasmic IGFBP-3 immunohistochemistry (IHC) scores were analysed as a continuum, with lymph node status and lymphovascular invasion assessed as present or absent. Significant *P *values (*P *< 0.05) are indicated in bold.

ER, oestrogen receptor.
